# Custom gene expression panel for evaluation of potential molecular markers in hepatocellular carcinoma

**DOI:** 10.1186/s12920-022-01386-7

**Published:** 2022-11-07

**Authors:** Srinivas Reddy Pallerla, Nghiem Xuan Hoan, Sivaramakrishna Rachakonda, Christian G. Meyer, Hoang Van Tong, Nguyen Linh Toan, Le Thi Kieu Linh, Dao Phuong Giang, Peter G. Kremsner, Mai Hong Bang, Le Huu Song, Thirumalaisamy P. Velavan

**Affiliations:** 1 Institute of Tropical Medicine, Universitätsklinikum Tübingen, Universität Tübingen, Wilhelmstr 27, 72074 Tübingen, Germany; 2 Vietnamese-German Center for Medical Research (VG-CARE), Hanoi, Vietnam; 3 Department of Molecular Biology, 108 Institute of Clinical Medical and Pharmaceutical Sciences, Hanoi, Vietnam; 4 Vietnam Military Medical University, Hanoi, Vietnam; 5 Centre de Recherches Medicales de Lambarene, Lambaréné, Gabon; 6 Faculty of Gastroenterology, 108 Institute of Clinical Medical and Pharmaceutical Sciences, Hanoi, Vietnam

**Keywords:** Hepatocellular carcinoma, HCC, Hepatitis B virus, Vietnam, Biomarker, Prognosis

## Abstract

**Background:**

Hepatocellular carcinoma (HCC) is the second leading cause of cancer-related mortality worldwide. It is a highly heterogeneous disease with poor prognosis and limited treatment options, which highlights the need for reliable biomarkers. This study aims to explore molecular markers that allow stratification of HCC and may lead to better prognosis and treatment prediction.

**Materials and methods:**

We studied 20 candidate genes (HCC hub genes, potential drug target genes, predominant somatic mutant genes) retrieved from literature and public databases with potential to be used as the molecular markers. We analysed expression of the genes by RT-qPCR in 30 HCC tumour and adjacent non-tumour paired samples from Vietnamese patients. Fold changes in expression were then determined using the 2^−∆∆CT^ method, and unsupervised hierarchical clustering was generated using Cluster v3.0 software.

**Results:**

Clustering of expression data revealed two subtypes of tumours (proliferative and normal-like) and four clusters for genes. The expression profiles of the genes *TOP2A, CDK1, BIRC5, GPC3, IGF2,* and *AFP* were strongly correlated. Proliferative tumours were characterized by high expression of the c*-MET, ARID1A, CTNNB1, RAF1, LGR5*, and *GLUL1* genes. *TOP2A, CDK1*, and *BIRC5* HCC hub genes were highly expressed (> twofold) in 90% (27/30), 83% (25/30), and 83% (24/30) in the tissue samples, respectively. Among the drug target genes, high expression was observed in the *GPC3, IGF2* and *c-MET* genes in 77% (23/30), 63% (19/30), and 37% (11/30), respectively. The somatic mutant Wnt/ß-catenin genes (*CTNNB1, GLUL and LGR5*) and *TERT* were highly expressed in 40% and 33% of HCCs, respectively. Among the HCC marker genes, a higher percentage of tumours showed *GPC3* expression compared to *AFP* expression [73% (23/30) vs. 43% (13/30)].

**Conclusion:**

The custom panel and molecular markers from this study may be useful for diagnosis, prognosis, biomarker-guided clinical trial design, and prediction of treatment outcomes.

**Supplementary Information:**

The online version contains supplementary material available at 10.1186/s12920-022-01386-7.

## Introduction

Hepatocellular carcinoma (HCC) is the second most common cause of cancer-related mortality worldwide, with two-thirds of cases occurring in Asia [1]. The hepatitis B (HBV) and hepatitis C (HCV) viruses are the major etiological agents for HCC globally [2], and in particular HBV-induced HCC is common with increasing numbers of cases in East Asia and Africa [3]. Vietnam has a high prevalence of HBV infections, ranging from 10 to 20% in the general population and 20–40% among injecting drug users and HIV-positive patients [4]. Prognosis of HCC remains poor because of the underlying chronic liver disease; late diagnosis, often at advanced stages of disease [5].

HCC is heterogeneous and classified into four Barcelona Clinic Liver Cancer (BCLC) stages (BCLC-A: early HCC, BCLC-B: intermediate HCC, BCLC-C: advanced HCC, BCLC-D: terminal HCC) for optimal clinical management using the staging depending on tumour burden, liver function (Child–Pugh score), and overall health [6, 7]. HCC is routinely diagnosed by blood tests (alanine transaminase [ALT], aspartate transaminase [AST], total bilirubin and direct bilirubin), liver biopsy, imaging, and serum alpha-fetoprotein (AFP) and albumin levels. HCC is heterogeneous, which is associated with poor prognostic outcomes because it is more difficult to diagnose accurately. Heterogeneity of HCC tumours are known to occur at different levels; firstly within the population and secondly within tumours of the same patient [8]. Recently, rapid progress has been made in understanding the heterogeneity of HCC by molecular subclassification using molecular and genetic markers [9]. HCC tumours are divided into two major phenotypic classes: the proliferation class and the non-proliferation class [10, 11], which have been identified on the basis of transcriptomic dysregulations and genetic alterations closely related to risk factors, pathologic features, and prognosis. This proliferation class is associated with HBV infection and has a poor clinical outcome. These are also subdivided into several molecular subclasses based on transcriptional profile, as discussed in extensively in several articles [11–16]. This classification would allow identification of predictive biomarkers and facilitate targeted treatments.

The pathogenesis of HCC is a multistep process involving progressive accumulation of genetic alterations in distinct oncogenes. Progression of HCC begins with chronic inflammation caused by viral (HBV, HCV) or non-viral factors that are known to alter the liver microenvironment. As a result, the liver cells increase the production of cytokines, reactive oxygen and nitrogen species that mediate liver injury and trigger the liver's regenerative response. This regeneration predisposes the hepatocyte cells to a variety of genetic alterations at the genomic and transcriptional levels leading to HCC [17–20]. The most commonly altered genetic variants in HCC include mutations of the *TERT* promoter, *CTNNB1, TP53, RB1, CCNA2, CCNE1, PTEN, ARID1A, ARID2, RPS6KA3* or *NFE2L2, CCND1, FGF19, VEGFA, MYC* and *MET* [21, 22], leading to changes in TERT, Wnt/β-catenin, p53/p21 and RB1, AKT-mTOR, RAS-MAPK, VEGF/VEGFR, MET, IGF, ARID1A/ARID1B/ARID2 pathways. Understanding the pathways and genes in HCC plays an important role in cancer prognosis and prediction of treatment success through stratification of cases for personalized treatment.

Despite some recent success in the development of new drugs approved for treatment of advanced HCC [23], several clinical trials have failed or shown only modest improvement in overall survival [23]. Further novel drug combinations and treatment regimens with first- and second-line drugs are currently under investigation [24]. As HCC is clinically and molecularly very heterogeneous, the use of biomarkers may guide personalized treatment strategies in clinical trials, leading to more favourable therapeutic outcomes. Recent developments in biomarker-driven therapies show some promise. To identify biomarkers associated with heterogeneous HCC tumours, several studies and multiple *insilico* computational bioinformatics analysis [25–29] using publicly available high-throughput HCC datasets have identified critical genes and pathways [17–20]. Some of the recent markers identified in the studies include; Cell division cycle-associated protein-3 (CDCA3) [30], ribophorins (RPNs), ARID1A [31]. These molecular markers of HCC enable to predict prognosis and to develop a new rationale for targeted therapeutic strategies. To date, several specific markers and key pathways involved in the HCC development have been identified potentially to assist early diagnosis, to predict prognosis and molecular targeted therapies of HCC tumours have been extensively reviewed. Recent developments in biomarker-driven therapies show some promises have been reviewed [32, 33]. For example, in a phase Ib/II HCC trial, patients with high *MET* mRNA expression showed a threefold increase in progression-free survival compared to patients with low *c-MET* expression when given a combination of the anti-VEGFR-2 mAb ramucirumab plus the anti-MET mAb emibetuzumab [34]. The gene expression profiles are used to identify differentially expressed genes (DEGs) during cancer progression [35, 36], which enables the identification of biomarkers for the prognosis, diagnosis, and targeted therapy of tumours [37].

To understand the heterogeneity of HCC and identify appropriate molecular markers, the present study examined a panel of genes including HCC hub, drug target, and somatic mutation genes with the potential to serve as meaningful biomarkers in prognosis and prediction of therapeutic success.

## Materials and methods

### Study population

The clinical and diagnostic characteristics of the study group are summarized in Table 1. To determine gene expression, pairs of HCC tumour (T) and adjacent non-tumour (NT) tissue specimens were obtained from 30 HCC patients who underwent surgery at the Vietnam National Cancer Hospital, Tan Trieu, Ha Dong in Hanoi in 2018. All patients were negative for anti-HCV and anti-HIV antibodies, nor had they a history of drug abuse. Liver function tests including ALT, AST, total bilirubin and direct bilirubin, albumin and prothrombin were quantified. HCC patients were categorized according to the BCLC staging system [38, 39]. All blood and T and NT tissue samples were stored at − 80 °C until further use.

**Table 1:** Baseline characteristics of 30 hepatocellular carcinoma (HCC) patients

Characteristics	n (%)
Age (years)	
< 40	3 (10)
40–60	17 (57)
> 60	10 (33)
Gender	
Male	25 (83)
Female	5 (17)
Aetiologies	
Hepatitis B virus (HBV)	26 (87)
Non-HBV/HCV	4 (13)
Barcelona Clinic Liver Cancer (BCLC) classification	
Stage A	1 (3)
Stage B	29 (97)
Metastasis	
Yes	2 (7)
No	28 (93)
Size of tumor (cm)	
< 3	1 (3)
3–5	14 (47)
≥ 5	15 (50)
Number of tumors	
1	23 (77)
2	4 (13)
≥ 3	3 (10)
Clinical parameters	Median (range)
Alpha Fetoprotein (ng/mL)	20 [2–19,724]
Carbohydrate Antigen 19-9 (U/L)	3 [0.7–13]
Carcinoembryonic Antigen (ng/mL)	12 [0.6–104]
HBV-DNA	NA
White blood cells (× 10^3^/mL)	7 [4–46]
Red blood cells (× 10^6^/mL)	5 [3–8]
Platelets (× 10^3^/mL)	185 [2–391]
Aspartate amino transferase (IU/mL)	37 [11–258]
Alanine amino transferase (IU/mL)	67 [17–242]
Total Bilirubin (µmol/L)	12 [5–72]
Direct Bilirubin (µmol/L)	4 [2–49]
Prothrombin (% of standard)	94 [39–115]
Protein (g/L)	75 [63–84]
Albumin (g/L)	41 [5–49]

*IU* International unit, *NA* Not analysed

### Serum levels assessment of AFP, CEA and CA 19-9

Serum levels of the markers AFP, CEA, and CA19-9 were measured using the ARCHITECT AFP reagent kit, Cat. No. 03P3625 (Abbott Ireland Diagnostics, Ireland), ARCHITECT CEA reagent kit, Cat. No. 07K6832 (Abbott Ireland Diagnostics, Ireland), and ARCHITECT CA 19-9XR Reagent Kit, Cat. No. 2K91 (Abbott GMBH & Co. Germany) respectively on the ARCHITECT i2000sr—an automated immunoassay analyser (Abbott Diagnostics, Abbott Park, IL, USA) according to the manufacturer's protocol.

### Genes selection criteria for the qPCR panel

The custom qPCR panel included 20 genes (Table 2) retrieved from scientific literature with biomarker potential for diagnosis, prognosis, and prediction of treatment outcomes. Specifically, genes belonging to the categories of HCC hub genes (prognosis and diagnostic potential), drug target genes and somatic mutated genes were considered. Another inclusion criterion was that we considered genes whose expression was significantly upregulated in at least 30–40% of HCCs (www.proteinatlas.org).

**Table 2:** Hepatocellular carcinoma qPCR panel genes: diagnosis, prognosis, and treatment prediction

Genes	Pathway	Criteria for selection	Source
HCC hub genes			
*TOP2A* (DNA topoisomerase 2-alpha)	Cell cycle	Poor prognosis and overall survival	[26, 27, 37, 40]
*CDK1* (cyclin-dependent kinase 1)	Cell cycle	Poor prognosis and overall survival	[37]
*BIRC5* (baculoviral IAP repeat-containing 5)	Cell cycle	Poor prognosis and overall survival	[41]
*CDC20* (cell division cycle protein 20 homolog)	Cell cycle	Poor prognosis and overall survival	[26, 37]
Drug target genes			
*GPC3* (glypican-3)	Extracellular molecule	Diagnostic marker and drug target	[42, 43]
*IGF2* (insulin-like growth factor 2)	AKT/mTOR; RAS/MAPK	Drug target	[44, 45]
*c-MET* (c-MET proto-oncogene, receptor tyrosine kinase)	AKT/mTOR; RAS/MAPK	Drug target	[44, 46]
*IDH1* (Isocitrate dehydrogenase 1)	TCA cycle	Drug target	[47]
*MCL1* (myeloid cell leukemia-1)	Apoptotic	Drug target	[48]
*MDM4* (MDM4 regulator of P53)	P53	Drug target	[49]
*RAF* (proto-oncogene c-RAF)	Ras/RAF	Drug target	[50]
*VEGFA* (vascular endothelial growth factor A)	VEGF	Drug target	[51]
*PD-L1* (programmed cell death ligand 1)	Immune checkpoint	Drug target	[44]
Somatic mutated and/ drug target genes			
*CTNNB1* (catenin beta 1) [*GLUL1* (glutamine synthetase) and *LGR5* (G-protein–coupled receptor)]	Wnt/ß-catenin	Somatic mutations, drug target and overall survival	[52–54]
*TERT* (telomerase reverse transcriptase)	Telomere maintenance	Somatic mutations, drug target and overall survival	[52, 55]
*ARID1A* (AT-rich interactive domain-containing protein 1A)	Epigenetic modifier	Somatic mutations	[52, 56]
*TP53* (tumor protein p53)	Cell cycle	Somatic mutations	[52, 57]
Housekeeping and marker genes			
*AFP* (α-fetoprotein)	NA	HCC serum diagnostic marker	NA
*GAPDH* (glyceraldehyde 3-phosphate dehydrogenase)*, ACTB* (actin beta) and *TBP* (TATA-binding protein)	NA	Housekeeping genes	NA

*NA* Not applicable

### RNA isolation and real-time qPCR

Total RNA was extracted from approximately 5 mm^3^ of HCC tumor or adjacent normal liver tissue using 1 mL TRIzol™ reagent (Thermo Fisher Scientific, Waltham, MA, USA). Next, the tissues were homogenized with a Dounce homogenizer, and RNA was isolated according to the manufacturer’s instructions. The RNA pellet was resuspended in 30–50 µL of RNA-free water, depending on the pellet's size. Next, quality and quantity were assessed using a Nanodrop spectrophotometer (Thermo Fisher Scientific, Waltham, MA, USA). For cDNA synthesis, one microgram of total RNA from each sample was reverse transcribed in 20 µL reaction using the RevertAid First Strand cDNA Synthesis Kit (Thermo Fisher Scientific, Waltham, MA, USA) according to the manufacturer’s instructions. The cDNA was diluted to 1:10 with nuclease-free water and subjected to quantitative real-time PCR.

The quantitative real-time PCR was performed with the LightCycler 480 Instrument II (Roche, Basel, Switzerland) in 96 well plates using a 10 µL final volume containing 0.3 µmol/L of forward and reverse primers, 2 µL of diluted cDNA and 1X SensiFAST™ SYBR® No-ROX Kit (Bioline, Memphis, TN, USA). PCR cycle parameters were initial denaturation at 94 °C for 2 min, 40 cycles at 95 °C for 10 s, and 60 °C for 25 s, with the fluorescence signal acquired at the end of each cycle. A melting curve analysis was performed at the end of the amplification. All 23 genes (20 genes under investigation plus 3 housekeeping genes) were quantified in duplicates in 96 well plates having a couple of non-template reactions with *ACTB* primers. The primer sequences used are given in the Additional file 1: Table S1.

### Data analysis

The fold change gene expression between the tumour (T) and adjacent non-tumour (NT) pair was determined using the relative quantification 2^−∆∆CT^ method [58]. The fold change with ± two-fold to the transcript levels between T and adjacent NT was considered differentially regulated. For having a better control of the results, mean cycle threshold (Ct) of the three reference genes was taken for the normalization: Ct [ref] = mean (Ct [*GAPDH*], Ct [*ACTB*], Ct [*TBP*]). Correlations between genes were measured using Pearson correlation (r) and differences between groups were determined by either two-tailed t-test or ANOVA. The gene expression heatmap was generated using the Cluster v3.0 software (http://bonsai.hgc.jp/~mdehoon/software/cluster/software.html) [59]. The normalized gene-expression profiles of 30 samples were subjected to unsupervised hierarchical clustering for similarities in expression data. Hierarchical clustering was applied to both rows and columns. The output file was visualized in JavaTreeview (https://sourceforge.net/projects/jtreeview/) [60].

## Results

### Patients baseline characteristics and blood indices

Paired samples of T and NT tissues from 29 patients with BCLC-B classification and one patient with BCLC-A classification were analysed for the expression of the genes under investigation. The baseline characteristics of the patients are summarized in Table 1. Ninety percent were > 40 years of age at the time of HCC diagnosis, and the mean age of study participants was 57 ± 12 years; 83% were men. Chronic HBV infection was the predominant incidental factor of HCC development (87%). The remaining cases (13%) were due to unknown non-HBV and non-HCV factors. Among other risk factors, liver cirrhosis was found in 23% of patients, and smoking/alcohol consumption was observed in 57% of patients. Tumours in the right liver lobe (67%) were more frequent than in the left liver lobe (33%). Large (≥ 5 cm) tumours were detected in 50% of the cohort, an intermediate size (3–5 cm) in 47% and one patient (3%) had a tumour of < 3 cm diameter. Blood tests showed elevated ALT (> 40 U/L) and AST (> 56 U/L) in 30% and 13% of patients respectively, elevated total (> 17 µmol/L) and direct bilirubin (> 5 µmol/L) levels in 17% and lowered platelet counts (< 150 × 10^3^/mL) in 20% of the cohort.

### Identification of molecular HCC subtypes by gene expression profiling

**Fig. 1:**
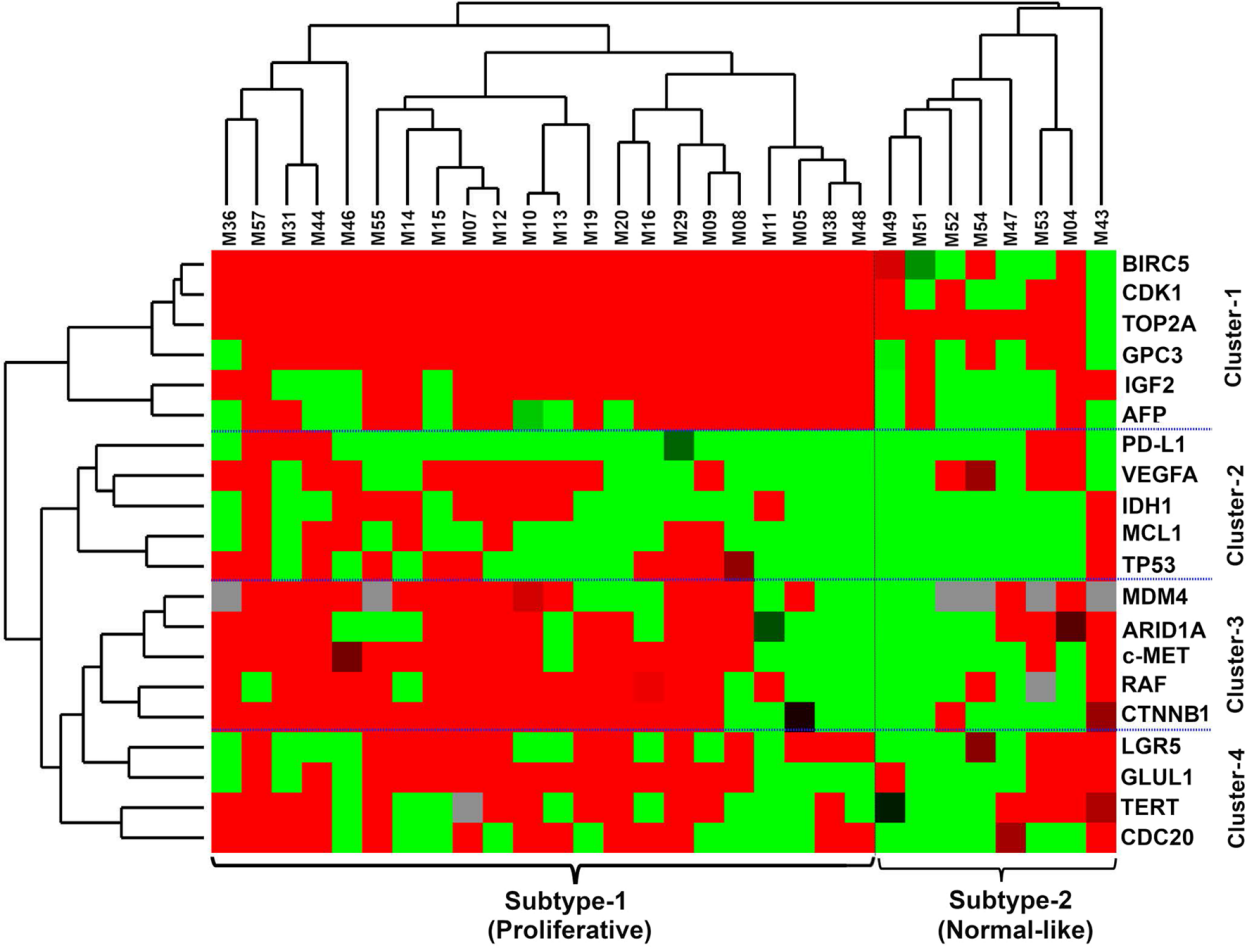
Heatmap shows gene expression Z-scores of differentially expressed mRNA of 20 genes in 30 paired HCC tumour samples. Clustering shows two subtypes of tumours (proliferative and normal-like) and four clusters of genes (Cluster-1 to Cluster-4)

Expression patterns of 20 genes were analysed in 30 paired tumour and non-tumour (T and NT) samples by RT-qPCR. Unsupervised hierarchical clustering using Manhattan distance and complete linkage revealed two subtypes of tumours (Fig. 1). Of the two subtypes, subtype-1 (proliferative) showed higher expression of genes compared to subtype-2 (normal-like). The proliferative subtype included 22 tumour samples compared eight for the normal-like subtypes.

**Fig. 2:**
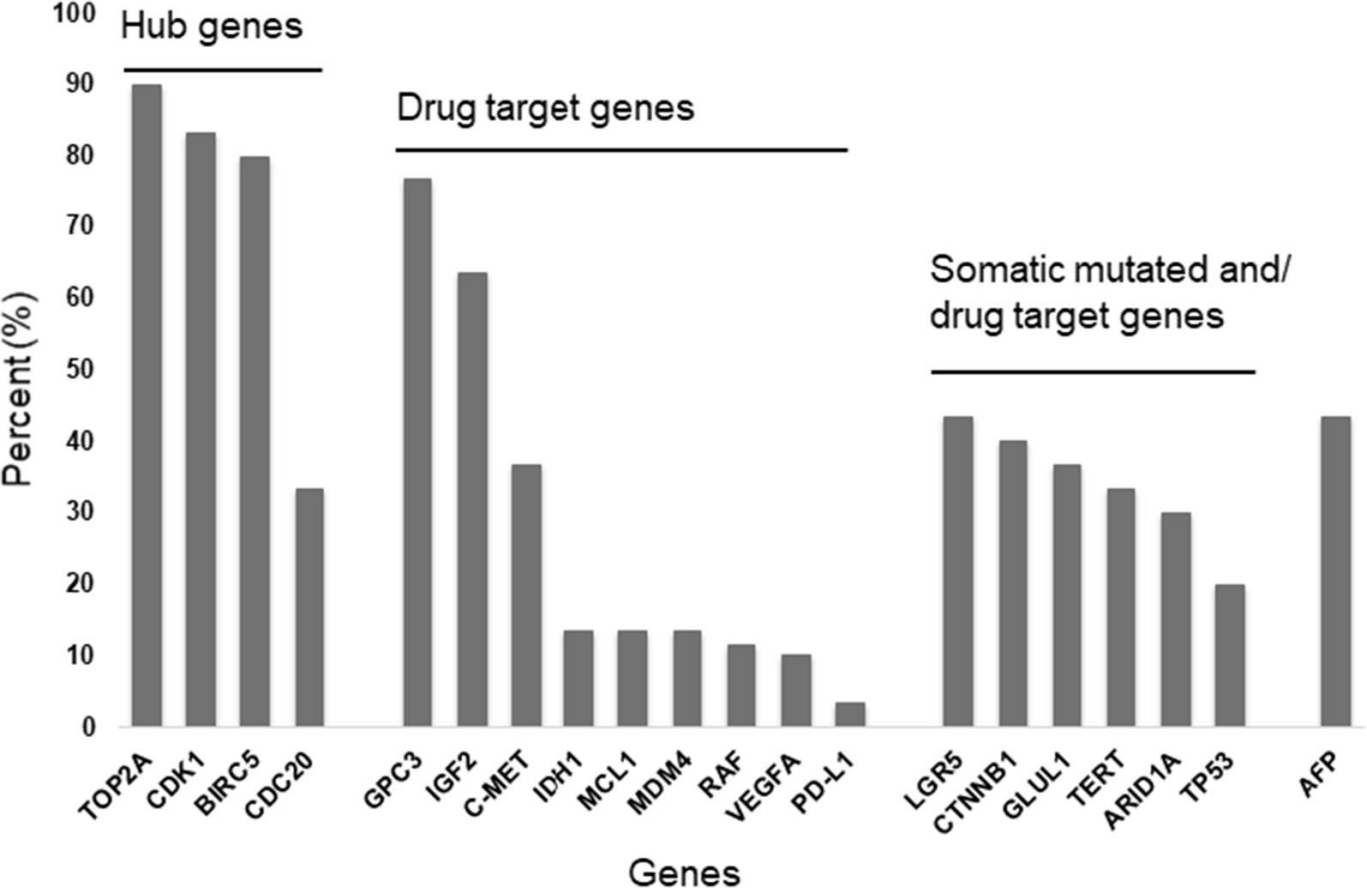
Percentage of HCC tumours in which gene expression levels of the 20 genes were > twofold overexpressed between tumour and non-tumour pairs

Clustering of the genes revealed four clusters. The hub genes *TOP2A, CDK1,* and *BIRC5* formed the top cluster with expression in 80–90% of the tumours. In addition to the hub genes, *GPC3, IGF2,* and *AFP* also showed uniform expression in both the proliferative, but not in the normal-like subtype. Expression of *AFP* showed a good correlation with the corresponding AFP serum levels (r:0.63, *p* < 0.001). The second cluster comprised of the *PD-L1, VEGFA, IDH1, MCL1* and *TP53* genes, which were diffusely upregulated in the proliferative subtype. The third cluster contained *MDM4, ARID1A, c-MET, RAF,* and *CTNNB1*, which were strongly upregulated in the proliferative subtype. The last cluster consisted of *LGR5, GLUL1, TERT* and *CDC20*, which were diffusely expressed in all subtypes. The bar graph represents the percentage of tumours from the highest to the lowest number of hub genes, drug target genes, and common somatic mutant genes that had > twofold gene expression (Fig. 2).

### Correlation between genes

The expression profiles of genes in the first cluster across all tumours (*TOP2A, CDK1, BIRC5, GPC3, IGF2* and *AFP*) were strongly correlated. The correlations between *TOP2A* and *CDK1* (r:0.77, *p* < 0.0001), *CDK1* and *BIRC5* (r;0.77, *p* < 0.0001) and *TOP2A* and *BIRC5* (r:0.65, *p* < 0.0001) were significant. The correlations between *GPC3* and *IGF2* (r:0.61, *p* < 0.0001) and between *GPC3* and *AFP* (r:0.78, *p* < 0.00001) and between *GPC3* and *IGF2* (r:0.55, *p* < 0.0001) were also significant.

The correlation between *TOP2A* and *CDK1* remained significant in both the tumour subtypes (r:0.58, *p*:0.004; r:0.79, *p*:0.02 in proliferative, and normal-like subtypes, respectively). The correlation between *TOP2A* and *BIRC5* (*r*:0.57, *p*:0.006), *TOP2A* and *GPC3* (*r*:0.48, *p*:0.02) was significant in the proliferative, but not in the normal-like subtype. Expression data for *MDM4* were available for 24 of the 30 tumours and its correlation with *TP53* was significant in proliferative (r:0.52, *p*:0.04), but not in normal-like (r:0.36, *p*:0.5). In the second gene cluster *(IDH1, VEGFA, MCL1*, *PD-L1, TP53*), statistically significant correlations were observed in proliferative subtype for *VEGFA* and *IDH1* (r:0.42, *p*:0.05), *VEGFA* and *MCL1* (r:0.43. *p*:0.05), *VEGFA* and *TP53* (r:0.52, *p*:0.01), *MCL1* and *IDH1* (r:0.57, *p* < 0.01), *PD-l1* and *TP53* (r:0.57, *p* < 0.01). In the normal-like subtype, correlations were weak between genes and not statistically significant. In the third cluster, the correlations between *c-MET* and *ARID1A* (r:0.74, *p* < 0.01), *c-MET* and *CTNNB1* (r:0.71, *p* < 0.01), *CTNNB1 and ARID1A* (r:0.51, *p*:0.03) and *LGR5* and *GLUL1* (r: 0.69, *p*:0.02) were only significant for the proliferative, but not in normal-like subtype. The correlation of *TERT* with *CDC20* was significant in both the subtypes (r:0.60, *p* < 0.01; r:0.80, *p*:0.02 in proliferative and normal-like subtypes, respectively).

### Comparison of tumour subtypes

The tumour sizes and serum levels of AFP, CEA and CA19-9 were compared between the proliferative and normal-like subtypes. Comparison of tumour sizes showed that the normal-like subtype showed larger tumour sizes in the normal-like (median [IQR]: 6.5 cm [3.8–8.5]) compared to the proliferative (4.8 cm [3–7]) and the difference was not statistically significant (t-test, two-tailed *p*:0.19).

**Fig. 3:**
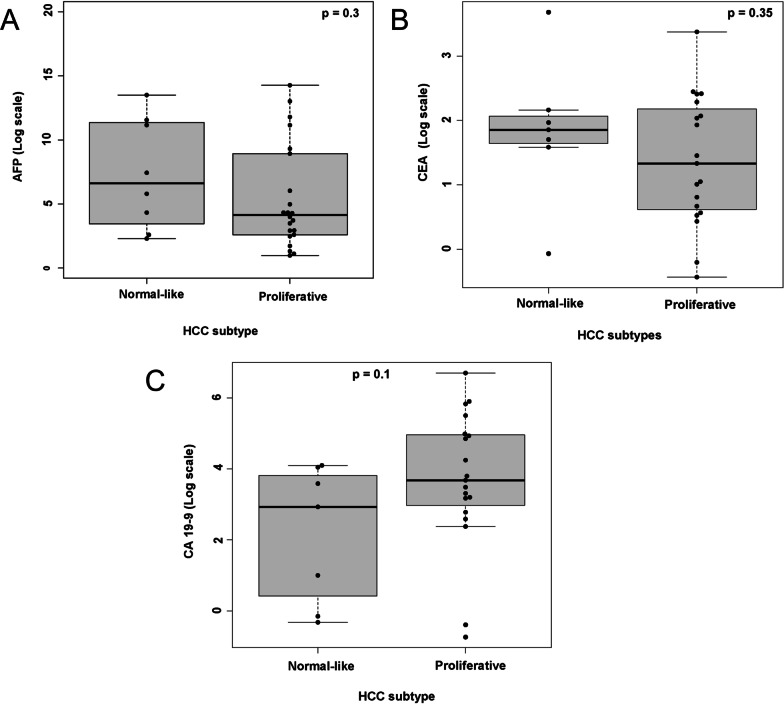
Comparison of serum AFP, CEA and CA19-9 levels (log-transformed) between proliferative and normal-like hierarchical subtypes

Comparison of serum AFP levels revealed higher levels in the normal-like subtype (median 114 ng/ml [16–2462]; (log_2_-transformed median 6.6 [7–11]) compared with the proliferative (median 16 ng/ml [6–486]; (log_2_-transformed median 4.1 [3–8])). No statistical difference in serum AFP levels was observed between the groups (*p*:0.30) (Fig. 3A). The normal-type subtype had higher levels of the serum marker CEA (median 3.6 ng/ml [3–4]; (log_2_-transformed median 1.8 [1.6–2])) compared to the proliferative subtype (median 2.3 ng/ml [1.5–4]; (log_2_-transformed median 1.3 [0.6–2])), although the difference between groups was not statistically significant (*p*:0.35) (Fig. 3B). The proliferative subtype showed higher serum CA19-9 levels (median 13.4 U/L [9–31]; (log_2_-transformed median 3.7 [3–5])) compared with normal-like (median 7.6 U/L [1.5–14]; (log_2_-transformed median 2.9 [0.4–4])), but no statistical difference was found between groups (*p*:0.1) (Fig. 3C).

## Discussion

The intratumoural heterogeneity among the BCLC-B patients is a major impediment in effective HCC therapy and renders long-term prognoses difficult and largely unreliable. The expression profile of key mutated genes and molecular classification of HCC and a subclassification for BCLC-B into four stages (B1-B4) has been proposed for better patient management. However, contradictory results from validation studies have shown a greater need of improved scoring systems and clinically relevant biomarkers [61–65]. To address the issue of biomarkers, our study investigated the expression pattern of important genes in HCC tumours and assessed their utility as biomarkers.

By using hierarchical clustering of gene expression data our study revealed two tumour subtypes: a proliferative and normal-like subtype. The proliferative subtype showed mutually exclusive expression patterns, except for the hub genes *GPC3, IGF2* and *AFP*. This observation is in agreement with findings published previously [44, 66, 67]. The proliferative subtype accounts for 50% of HCC cases and is associated with a highly aggressive phenotype and poor outcome [8]. In our data, the proliferative subtype showed higher expression of the *c-MET, ARID1A, CTNNB1, RAF1, MADM4, LGR5, GLUL1, TERT* and *CDC20* genes. Based on the expression profiles it is plausible that the RAS/MAPK, MET, AKT/mTOR and liver-Wnt pathways are responsible for cellular plasticity in the proliferative subtype [8, 44]. The normal-like subtype consisted of eight tumours and showed a scattered expression of hub genes (except *TOP2A*), drug-target genes and somatically mutated genes without any pattern, perhaps an indication that tissue analysed was mixed with normal tissue.

The higher expression of the hub genes *TOP2A, CDK1* and *BIRC5* in all samples indicates active cell division and proliferation. The selection of hub genes for the present study was based on their strong association with poor prognosis and overall survival in HCC patients [26, 27, 37, 40]. The strong correlation of *TOP2A* with *CDK1* and *BIRC5* as well as an earlier report showed that *TOP2A* could substitute other hub genes to be a good biomarker [37]. Moreover, *TOP2A* is associated with tumour grades, HBV infection, and vascular invasion [29]. In addition to expression of the hub genes, we noticed that *GPC3* and *IGF2* were expressed in 65–75% and *AFP* in more than 40% of the tumour samples. Our findings of *GPC3* expression in 75% of HCC cases confirms earlier reports of its utility as a biomarker [42, 68]. GPC3 enhances cell proliferation through the Wnt/β-catenin pathway and is associated with a poor prognosis [42, 68]. *IGF-2* expression is almost absent in adult hepatic cells, but its upregulation in HCC has been attributed to epigenetic mechanisms [69]. GPC3 and IGF-2 are potential drug targets that have significantly reduced tumour growth and prolonged survival in Phase 1 clinical trials and in animal models respectively [70, 71]. Overall, we show that, in addition to HCC hub genes, *GPC3 and IGF-2* may also serve as drug targets and early diagnostic markers for HCC.

The proliferative subtype in our study was characterised by high expression of the *c-MET, ARID1A, CTNNB1, RAF1, MDM4, LGR5* and *GLUL1* genes. Of these, *c-MET* along with hepatocyte growth factor (HGF) activates RAS-ERK and PI3K-AKT pathways, strengthening tumour aggressiveness with poor prognosis [72–74]. *ARID1A* has a context-dependent oncogenic and tumour suppressive function in HCC. A higher expression as seen in our study indicates oncogenic activity, supposedly mediated through cytochrome P450 and oxidative stress [75]. *RAF1* is a proto-oncogene that encodes MAP3 kinase and triggers cell proliferation through the ERK signalling pathway. Higher expression of *RAF1* is associated with resistance to sorafenib, a tyrosine-kinase inhibitor and a standard drug for the treatment of advanced HCC [76]. *CTNNB1* encodes beta-catenin which is essential for the canonical Wnt pathway; higher expression of this gene in HCC is mediated through exonic mutations and epigenetic factors [77, 78]. *LGR5* encodes a member of the G-protein coupled receptor superfamily and is believed to promote HCC metastasis formation by inducing epithelial-mesenchymal transition. *GLUL1* catalyses the synthesis of glutamine from glutamate and ammonium in the liver tissue and is tightly controlled by Wnt/β-catenin signalling. Mutations in *CTNNB1* activate the pathway leading to higher levels of *GLUL1* and *LGR5* [79, 80]. Overexpression of *GLUL1* sensitizes HCCs to sorafenib, indicating the relevance of *GLUL1* as a potential biomarker for the stratification of patients regarding treatment with sorafenib [81]. The other two genes that showed expression in proliferative subtype tumours are *TERT* and *CDC20*. *TERT* is responsible for maintaining telomere length in tumours and re-activated in HCCs primarily through mutations in promoter region [82]. Studies have shown a good concordance between mutations in CTNNB1 and the TERT promoter in HCCs, indicating that inhibitors targeting Wnt/β-catenin and TERT could be beneficial in HCC therapy [83]. *CDC20* regulates cell division through its interaction with anaphase-promoting complex/cyclosome (APC/C) and its overexpression is associated with poor prognosis [84].

Serum AFP, CA19-9, and CEA are used as preoperative tumour markers [85]. Especially, AFP is commonly used in clinical practice to diagnose HCC and various tumours [86–88]. In combination with AFP, serum markers CA19-9 and CEA are being used to improve the diagnostic and prognostic performance of HCC patients. In this study, comparing serum AFP levels between the tumour subtypes showed that the normal-like subtype showed trend with higher AFP serum levels compared to the proliferative. However, it was statistically insignificant. This contrasts with published results, namely that the proliferative subtype has higher AFP levels [44]. Interestingly, we observed a statistically significant positive correlation between *AFP* expression and AFP serum levels across all subtypes. Higher AFP levels promote HCC cell growth by activating the NF-κB pathway, in addition to suppressing the Fas/FADD-mediated apoptotic pathway [89, 90]. Higher AFP levels showed an association with higher metastasising activities and post-operative recurrence rates. In addition, we did not detect significant differences in serum levels of CA19-9 and CEA between proliferative and normal HCC subtypes.

## Conclusion

Taken together our study has shown two main subtypes in BCLC-B classified tumours. We have demonstrated that a molecular classification of HCC can be achieved through a gene panel using RT-PCR. This approach enables patient stratification based on gene expression profiles for targeted personalized treatment. The genes *c-MET, ARID1A, CTNNB1* and *RAF1* showing an association with the proliferative subtype in our study may be used as molecular markers for subtype determination, and hub genes can be applied for HCC diagnosis. However, large prospective, well-designed follow-up studies are required to evaluate these marker genes for clinical applications. Besides being a retrospective study, with a rather small sample size our major limitation has been lacking longitudinal follow-ups of patients for a comprehensive survival analysis.

## Supplementary Information

**Additional file 1. Table S1.** List of genes and primers.

## Data Availability

The datasets generated and/or analysed during the current study are available in the Gene Expression Omnibus (GEO, http://www.ncbi.nlm.nih.gov/geo/), [GEO Submission (GSE191298)].

## Abbreviations

HCC: Hepatocellular carcinoma
HBV: Hepatitis B virus
HCV: Hepatitis C virus
RT-qPCR: Real-time quantitative PCR
BCLC: Barcelona Clinic Liver Cancer
T: Tumour
NT: Non-tumour
ALT: Alanine transaminase
AST: Aspartate transaminase
AFP: Alpha fetoprotein
CEA: Carbohydrate antigen 19-9
CA 19-9: Carcinoembryonic antigen
HGF: Hepatocyte growth factor
ANOVA: Analysis of variance
