# Supplementary material for: Custom gene expression panel for evaluation of potential molecular markers in hepatocellular carcinoma
Source: BMC Med Genomics. 2022 Nov 7;15:235. doi: 10.1186/s12920-022-01386-7 (PMC9641913; doi:10.1186/s12920-022-01386-7)
Supplement: Supplementary file 1 — Additional file 1. Table S1. List of genes and primers. [file 12920_2022_1386_MOESM1_ESM.docx]

**Supplementary Table 1**: List of genes and primers

| **Genes** |  | **Primer sequence (5’-3’)** | **References** |
| --- | --- | --- | --- |
| *GAPDH* | F | NA | Thermo Fischer Cat# K1621 |
|  | R | NA |  |
| *ACTB* | F | CACCAACTGGGACGACAT | [1] |
|  | R | ACAGCCTGGATAGCAACG |  |
| *TBP* | F | ATCTTTGCAGTGACCCAGCATC | This study |
|  | R | TGCGCTGGAACTCGTCTCAC |  |
| *AFP* | F | GGGTGTTTAGAAAACCAGCTACC | This study |
|  | R | GCTGCAGCAGTCTGAATGTCC |  |
| *TERT* | F | AGTATGGCTGCGTGGTGAACT | This study |
|  | R | GGTCCGGGCATAGCTGGAGTA |  |
| *TP53* | F | GACACGCTTCCCTGGATTGG | This study |
|  | R | ACGGCAAGGGGGACAGAAC |  |
| *CTNNB1* | F | TCTGAGGACAAGCCACAAGATTACA | [2] |
|  | R | TGGGCACCAATATCAAGTCCAA |  |
| *GLUL1* | F | CCTGCTTGTATGCTGGAGTC | [3] |
|  | R | GATCTCCCATGCTGATTCCT |  |
| *LGR5* | F | TCAGTCAGCTGCTCCCGAAT | [4] |
|  | R | CGTTTCCCGCAAGACGTAAC |  |
| *ARID1A* | F | ATCAGTTCTCCACCCAAGGCAC | This study |
|  | R | TGGCCGCTTGTAATTCTGCTGTTG |  |
| *TOP2A* | F | TGAAGAAGACAGCAGCAAAAAGTCAGT | [5] |
|  | R | AAAATTAGAGTCAGAATCATCAGAAGTGG |  |
| *CDK1* | F | AGAGCGACGCGGTTGTTGTA | This study |
|  | R | TGGGTATGGTAGATCCCGGC |  |
| *BIRC5* | F | CATTCGTCCGGTTGCGCTTTC | This study |
|  | R | GCGCACTTTCTCCGCAGTTTC |  |
| *CDC20* | F | CGGAAGACCTGCCGTTACATTC | [6] |
|  | R | CAGAGCTTGCACTCCACAGGTA |  |
| *RAF1* | F | GCAGTTTGGCTATCAGCGCC | This study |
|  | R | CCATTTCGCACATTGACCACTGTTC |  |
| *C-MET* | F | GAAGAGGGCATTTTGGTTGTG | This study |
|  | R | CTCGGTCAGAAATTGGGAAAC |  |
| *IGF2* | F | GCTGTTCGGTTTGCGACACG | This study |
|  | R | GGATTCCCATTGGTGTCTGGAAG |  |
| *IDH1* | F | TTGGCTGCTTGCATTAAAGGTT | This study |
|  | R | GTTTGGCCTGAGCTAGTTTGA |  |
| *GPC3* | F | TGTCACCAAGTCCGCTCCTTCTTC | This study |
|  | R | CATGTTGGGCCCTTAGGGAGAC |  |
| *VEGFA* | F | GGGCAGAATCATCACGAAGT | [7] |
|  | R | CACACAGGATGGCTTGAAGA |  |
| *MCL1* | F | CCAAGAAAGCTGCATCGAACCAT | [8] |
|  | R | CAGCACATTCCTGATGCCACCT |  |
| *MDM4* | F | CGACTCATGGAGCTGCCGTAAG | This study |
|  | R | GAGAGATCCTGCAAGCACTGTCAG |  |
| *PD-L1* | F | GTGGCATCCAAGATACAAACTCAAAGAAGC | This study |
|  | R | CATTCCTTCCTCTTGTCACGCTCAG |  |

References:

1. Xiang X, Deng Z, Zhuang X, Ju S, Mu J, Jiang H, Zhang L, Yan J, Miller D, Zhang HG: **Grhl2 determines the epithelial phenotype of breast cancers and promotes tumor progression**. *PLoS One* 2012, **7**(12):e50781.

2. Zhang M, Shi J, Huang Y, Lai L: **Expression of canonical WNT/beta-CATENIN signaling components in the developing human lung**. *BMC Dev Biol* 2012, **12**:21.

3. Kung HN, Marks JR, Chi JT: **Glutamine synthetase is a genetic determinant of cell type-specific glutamine independence in breast epithelia**. *PLoS Genet* 2011, **7**(8):e1002229.

4. Bonnans C, Flaceliere M, Grillet F, Dantec C, Desvignes JP, Pannequin J, Severac D, Dubois E, Bibeau F, Escriou V *et al*: **Essential requirement for beta-arrestin2 in mouse intestinal tumors with elevated Wnt signaling**. *Proc Natl Acad Sci U S A* 2012, **109**(8):3047-3052.

5. Perea-Resa C, Bury L, Cheeseman IM, Blower MD: **Cohesin Removal Reprograms Gene Expression upon Mitotic Entry**. *Mol Cell* 2020, **78**(1):127-140 e127.

6. Zhang X, Morrissey C, Sun S, Ketchandji M, Nelson PS, True LD, Vakar-Lopez F, Vessella RL, Plymate SR: **Androgen receptor variants occur frequently in castration resistant prostate cancer metastases**. *PLoS One* 2011, **6**(11):e27970.

7. Shathasivam P, Kollara A, Spybey T, Park S, Clarke B, Ringuette MJ, Brown TJ: **VEPH1 expression decreases vascularisation in ovarian cancer xenografts and inhibits VEGFA and IL8 expression through inhibition of AKT activation**. *Br J Cancer* 2017, **116**(8):1065-1076.

8. Gholami O, Jeddi-Tehrani M, Iranshahi M, Zarnani AH, Ziai SA: **Mcl-1 is up regulated by prenylated coumarin, umbelliprenin in jurkat cells**. *Iran J Pharm Res* 2014, **13**(4):1387-1392.
